# A four‐gene signature associated with clinical features can better predict prognosis in prostate cancer

**DOI:** 10.1002/cam4.3453

**Published:** 2020-09-13

**Authors:** Penghui Yuan, Le Ling, Qing Fan, Xintao Gao, Taotao Sun, Jianping Miao, Xianglin Yuan, Jihong Liu, Bo Liu

**Affiliations:** ^1^ Department of Urology Tongji Hospital Tongji Medical College Huazhong University of Science and Technology Wuhan China; ^2^ Department of Geriatrics Tongji Hospital Tongji Medical College Huazhong University of Science and Technology Wuhan China; ^3^ Department of Oncology Tongji Hospital Tongji Medical College Huazhong University of Science and Technology Wuhan China

**Keywords:** clinical features, four‐gene signature, prognosis, prostate cancer

## Abstract

Prostate cancer (PCa) is one of the most deadly urinary tumors in men globally, and the 5‐year over survival is poor due to metastasis of tumor. It is significant to explore potential biomarkers for early diagnosis and personalized therapy of PCa. In the present study, we performed an integrated analysis based on multiple microarrays in the Gene Expression Omnibus (GEO) dataset and obtained differentially expressed genes (DEGs) between 510 PCa and 259 benign issues. The weighted correlation network analysis indicated that prognostic profile was the most relevant to DEGs. Then, univariate and multivariate COX regression analyses were conducted and four prognostic genes were obtained to establish a four‐gene prognostic model. And the predictive effect and expression profiles of the four genes were well validated in another GEO dataset, The Cancer Genome Atlas and the Human Protein Atlas datasets. Furthermore, combination of four‐gene model and clinical features was analyzed systematically to guide the prognosis of patients with PCa to a largest extent. In summary, our findings indicate that four genes had important prognostic significance in PCa and combination of four‐gene model and clinical features could achieve a better prediction to guide the prognosis of patients with PCa.

## Graphical abstract

Prostate cancer (PCa) is one of the most deadly urinary tumors in men globally, and the progression and development of PCa is associated with copious genetic aberrations. This study is aimed to add novel biomarkers of PCa development and prognosis by analyzing the genetic changes and clinical traits comprehensively. Based on integrated analysis, four genes were significantly related to the prognosis of PCa and well validated in other datasets. Furthermore, combination of four genes and clinical features achieved a better prediction to guide the prognosis of patients with PCa.

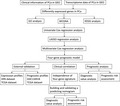

## 1 INTRODUCTION

Prostate cancer (PCa) is the second most commonly diagnosed and the fifth leading death of cancer in men worldwide.^1^ It is observed that the incidence of PCa has been growing globally, particularly in Asia, Northern and Western Europe.^2^ The metastasis of PCa is the dominant factor of PCa‐related death, resulting in the 5‐year mortality over 70%.^3^ As a significant sign of prognosis, biochemical recurrence (BCR), defined as the return of prostate‐specific antigen (PSA), occurs in 27%‐53% of patients after radical prostatectomy and radiotherapy,^4^ and tends to happen months or years ahead of other clinical symptoms of PCa recurrence ^5^ and increases the risk of developing distant metastases, PCa‐specific, and overall mortality.^6^ Although extensive research has been conducted on the mechanisms of carcinogenesis, the etiology of PCa still remains unclear. Therefore, it is significant to explore biomarkers for early diagnosis, prognosis, and personalized therapy of PCa.

Various molecular aberrations and genetic mutations exist in the pathogenesis of PCa.^7^, ^8^ In the meantime, cumulative studies have been conducted to identify novel molecular biomarkers and refine diagnosis and prognosis of PCa recently,^5^ nevertheless, not all molecular alternations influence the tumor outcome, and a single gene aberration does not necessarily have a good predictive value due to complex carcinogenesis and individual difference.^9^ It will be more valuable to explore multiple molecules combined with clinical features for better prediction of diagnosis and prognosis in PCa. Nowadays, analysis of microarray and high‐throughput sequencing technologies has advanced diagnosis and prognosis of various diseases.^10^ It provides an efficient tool to decipher critical molecular alternations especially in cancer. A comprehensive understanding of molecular patterns of PCa as well as clinical traits would contribute to identifying the prognostic risk of patients and achieving personalized therapy by a systematic model.

In this study, we performed an integrated analysis in multiple microarrays and identified hub genes affecting the prognosis. Then, a four‐gene prognostic model was constructed and patients of PCa were stratified based on risk score. The predictive value and gene profiles were validated successfully in other independent PCa datasets. Finally, combination of four‐gene model and clinical features was analyzed systematically to guide the prognosis of patients with PCa to a largest extent. In summary, the study is aimed to add novel knowledge of PCa development and prognosis by analyzing the genetic changes and clinical traits comprehensively.

## 2 MATERIALS AND METHODS

### 2.1 Data acquisition

Transcriptome data of PCa was obtained from the Gene Expression Omnibus (GEO) datasets (https://www.ncbi.nlm.nih.gov/geo) (GSE21034,^11^ GSE55945,^12^ GSE46602,^13^ GSE62872,^14^ and GSE29079 ^15^) on April 3, 2020. Each eligible dataset incorporated at least twenty samples of tumor and benign issue. After screening, 510 tumor samples and 259 benign samples were acquired for further analysis finally. This study were approved by our Institutional Research Ethics Committee.

### 2.2 Integrated analysis of microarray datasets

Raw transcriptome data in each dataset was normalized by “limma” package ^16^ in R software (Version 3.6.2). After averaging the expression values of the genes corresponding to the multi‐microarray probes and base‐2 logarithm (log2) transformation, log2 fold‐change (log_2_FC) values of differentially expressed genes (DEGs) expressions were calculated between tumor and normal issues by “limma” package. Later, the integrated analysis was conducted across the five microarray datasets by robust rank aggregation algorithm with R package “RobustRankAggregation” based on a prioritized gene list.^17^ DEGs with |log_2_FC| > 1 and adjust *P* < .01 were regarded as statistical significance for further analysis.

### 2.3 Functional enrichment analysis

To identify the biological functions related to DEGs, the Gene Ontology (GO) and Kyoto Encyclopedia of Genes and Genomes (KEGG) pathway enrichment analyses of DEGs were conducted by R package “clusterProfiler” ^18^ with the thresholds of adjusted *P* < .05 and *q* < 0.05. The GO and KEGG clusters were visualized by the R package “GOplot”.^19^

### 2.4 Evaluation of gene modules and correlation with clinical factors

To establish the gene interaction modules and assess the relationship between DEGs and clinical factors as a whole, the weighted correlation network analysis (WGCNA) was conducted for DEGs by R package “WGCNA”.^20^ Clinical data were extracted from GSE70769 dataset,^21^ which including clinical stage, T stage, Gleason score, prognostic conditions as well as gene expression profiles. The value of soft threshold (power) was set to obtain the optimal scale free topology fit model index (scale free *R* ^2^) and mean connectivity in the meantime. Based on a topological overlap measure to determine the degree of dissimilarity among genes, cluster dendrogram of genes was obtained. After clustering modules and genes, clinical factors in GSE70769 were involved in correlation analysis with the module eigengenes. *P* < .05 was regarded as statistical significance.

### 2.5 Construction and assessment of prognostic model based on DEGs

Differentially expressed genes were applied for prognostic analysis with clinical information in the GSE70769 dataset. First, univariate Cox regression method was employed to obtain candidate genes related to prognosis with *P* < .05 between patient BCR free survival and gene expression levels. Next, the LASSO Cox regression analysis by R package “glmnet”^22^ was applied to further screen the candidate genes with more prognostic value based on penalty parameter tuning performed via 10‐fold cross‐validation.^23^ Finally, the selected genes were involved in the multivariate Cox regression model by a stepwise method. After these, a risk score tool was acquired based on the sum of gene expression level (Exp*_i_*) and regression coefficient (*β_i_*) based on the multivariate Cox regression model (risk score = ΣExp*_i_* × β*_i_*). The patients with clinical information were classified into different risk groups according the medium value of risk score. Then, Kaplan‐Meier (KM) survival curves by R package “survival,” receiver operational characteristic (ROC) curves by R package “timeROC”^24^ and C‐index were processed to evaluate the prognostic value of the multigene model. Finally, the model was validated in another GEO dataset (GSE116918) including 248 patients and The Cancer Genome Atlas (TCGA) dataset including 499 patients (https://portal.gdc.cancer.gov/).^25^ And the prognostic value of the multigene model was also evaluated compared with T stage in GSE70769.

### 2.6 Verification of the expression profiles of prognostic genes

To validate the value of prognostic genes above, the expression profiles of these genes were analyzed in TCGA prostate adenocarcinoma cohort^26^ with the Mann‐Whitney *U* test. *P* < .05 was regarded as statistical significance. Furthermore, to determine the clinical relevance of these genes, immunohistochemical data were downloaded from the Human Protein Atlas (HPA) (https://www.proteinatlas.org/)^27^ to compare the levels of protein encoded by these genes. The results of immunohistochemistry in tumor and normal issues were shown by the same antibody.

### 2.7 Combination of prognostic genes and clinical data for prognostic evaluation

To assess the association between the prognostic genes and other clinical features (including risk score based on prognostic genes, Gleason score, preoperative level of PSA, clinical stage, extra capsular extension, and positive surgical margins) and construct a more systematic prognostic model, univariate and multivariate Cox regression analyses were processed with BCR free survival as the dependent variable and prognostic genes as well as clinical features as the independent variables. Hazard value and *P* value (<.05) were obtained for assessment of prognosis. Based on the results, a new model combining prognostic genes and significant clinical features was constructed. Similarly, the KM survival curves, ROC curves, and C‐index were shown. Furthermore, a nomogram was established to show prognostic profiles and calibration curves were drawn to verify the accuracy of the nomogram by R package “rms”.^28^

## 3 RESULTS

### 3.1 Identification of DEGs based on integrated analysis

**FIGURE 1:**
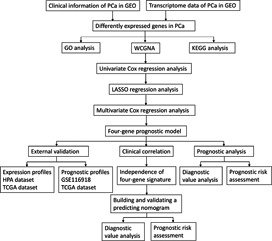
The flow diagram of the study. GEO, Gene Expression Omnibus; GO, Gene Ontology; HPA, Human Protein Atlas; KEGG, Kyoto Encyclopedia of Genes and Genomes; PCa, prostate cancer; TCGA, The Cancer Genome Atlas; WCGNA, weighted correlation network analysis

**FIGURE 2:**
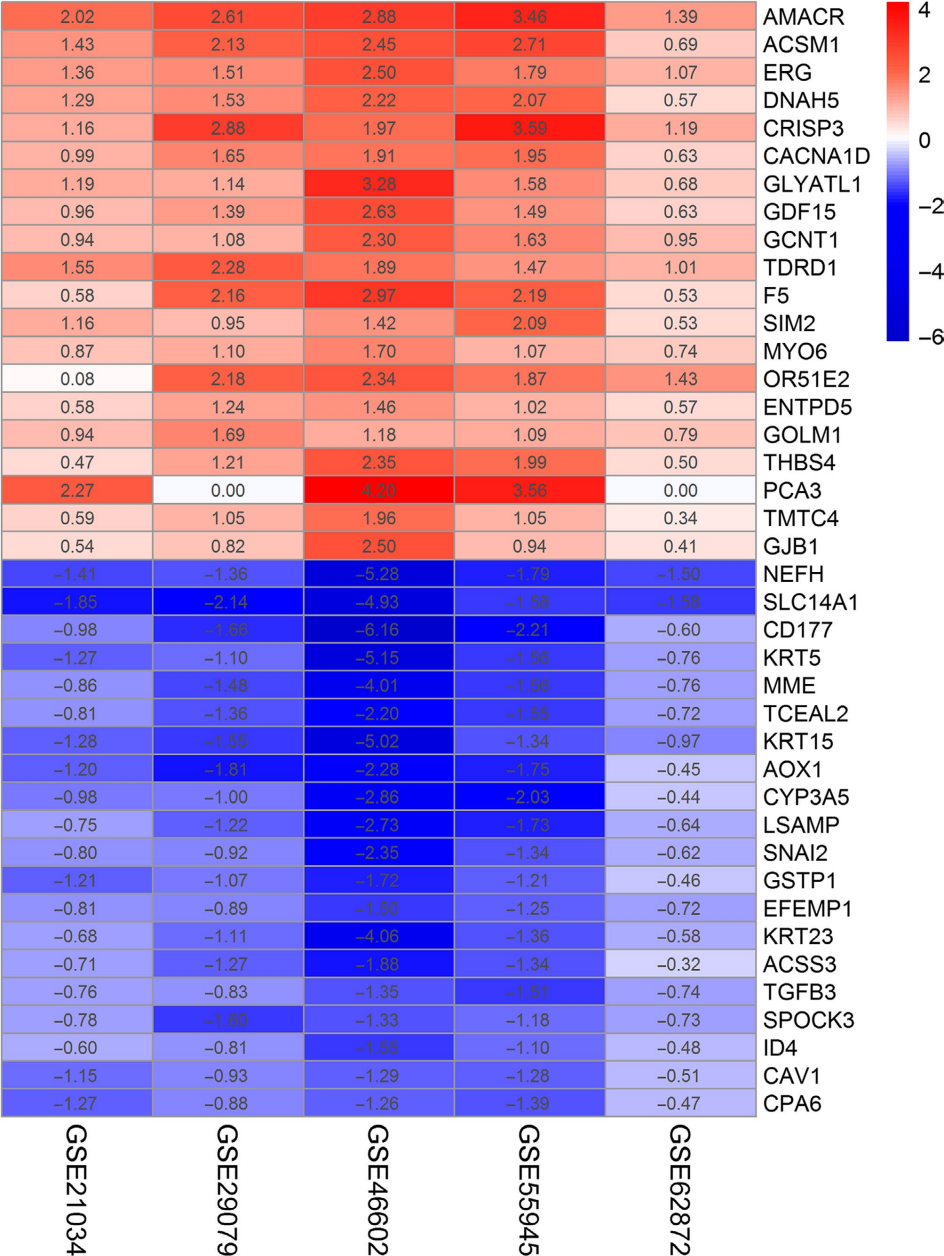
A heat map of the top 20 significantly upregulated and downregulated DEGs based on integrated analysis. The expression profile of genes in each dataset is shown by log_2_FC value in each column. The color layout from blue to red represents the expression level of DEGs from downregulation to upregulation. DEGs, differentially expressed genes; log_2_FC value, base‐2 logarithm transformation fold‐change value

The flow diagram in this study is illustrated in Figure 1. The information of five GEO datasets involved in this study is shown in Table S1. There were 510 PCa samples and 259 benign samples for further analysis. After normalization and integrated analysis in each dataset (Figure S1), a total of 270 DEGs involving 148 downregulated and 122 upregulated genes were identified by robust rank aggregation algorithm. The top 20 DEGs in both of them are shown in Figure 2. AMACR, ACSM1, ERG, DNAH5, and CRISP3 were top five genes in upregulated sets and NEFH, SLC14A1, CD177, KRT5, and MME were top five genes in downregulated sets.

### 3.2 Identification of biological functions related to DEGs

**FIGURE 3:**
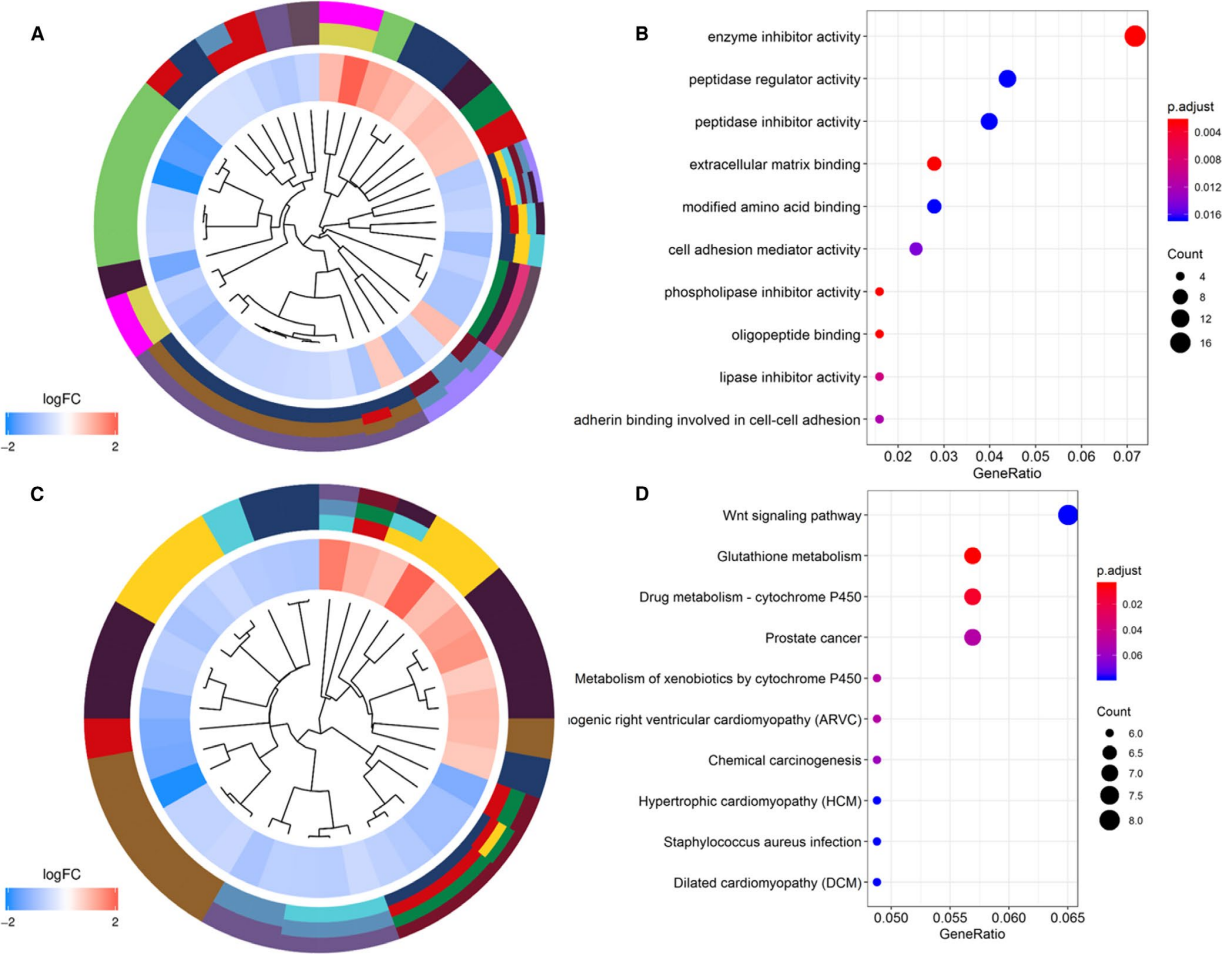
Functional enrichment analysis of DEGs. A, GO cluster. B, GO analysis of representative DEGs. The *y*‐axis shows enriched GO terms. C, KEGG cluster. D, KEGG analysis of representative DEGs. The *y*‐axis shows enriched KEGG pathways. For (A) and (C), the innermost part shows the hierarchical clustering of the DEGs. The middle part represents the expression profiles of DEGs, in which the color layout from blue to red indicates the expression level of DEGs from downregulation to upregulation. And the outermost part represents the GO terms (A) and KEGG pathways (C) associated with DEGs. DEGs, differentially expressed genes; GO, Gene Ontology; KEGG, Kyoto Encyclopedia of Genes and Genomes

GO and KEGG enrichment analyses were conducted to reveal the biological roles of DEGs. The GO profiles of DEGs are shown in Figure 3A. It revealed that these DEGs had a close relationship with metabolism and peptidase activities (Figure 3B and Table S2). The top five GO terms were enzyme inhibitor activity, peptidase regulator and inhibitor activities, modified amino acid binding, and extracellular matrix binding. Similarly, KEGG profiles are shown in Figure 3C. These DGEs mainly participated in Wnt signaling pathway, glutathione metabolism, metabolism of drug, and xenobiotics by cytochrome P450. Also, PCa pathway was involved significantly (Figure 3D and Table S3).

### 3.3 Weighted correlation network analysis

**FIGURE 4:**
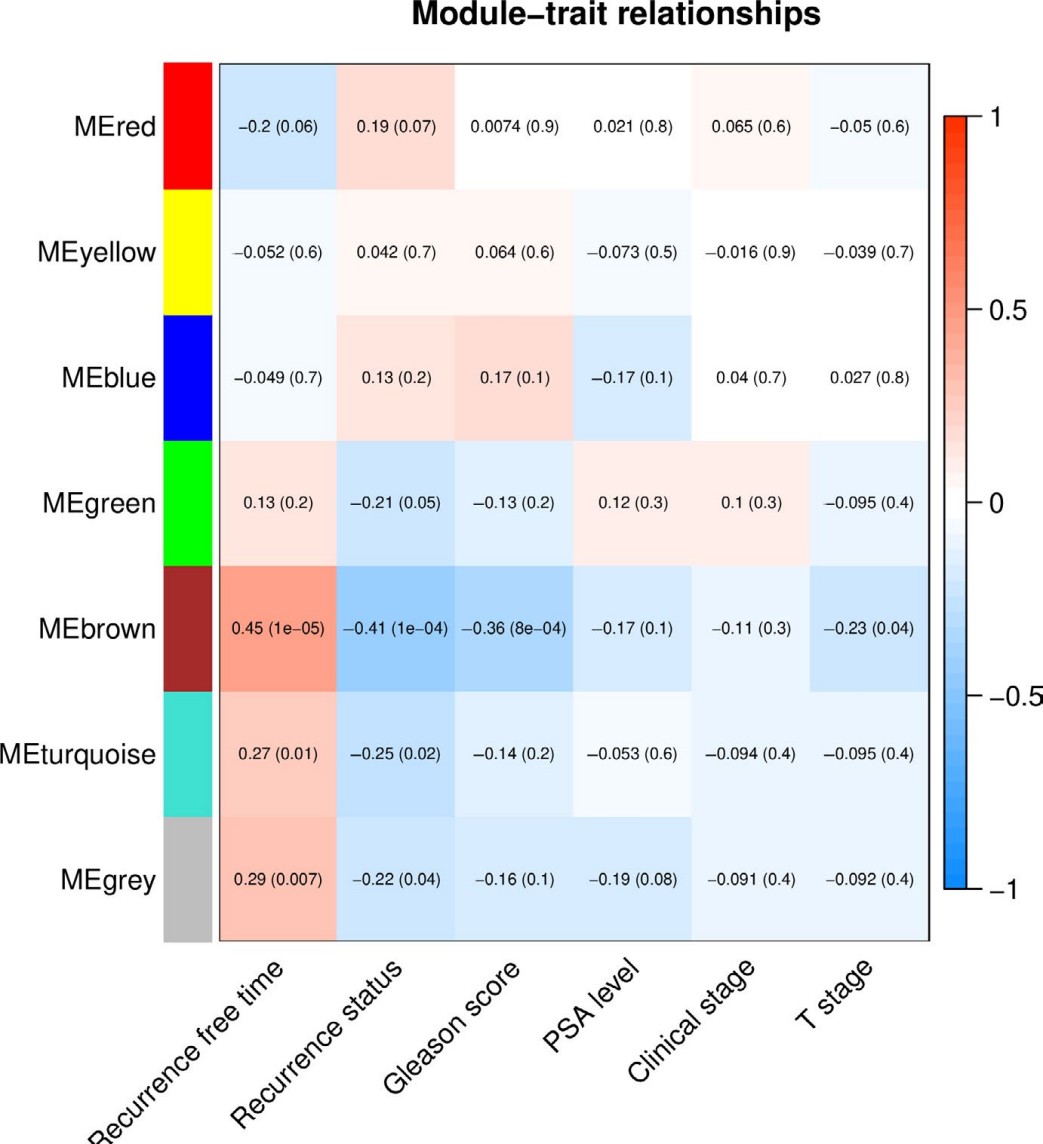
Module‐trait relationships based on WGCNA analysis. Each column represents a clinical trait and each row represents a gene module. The correlation coefficient and corresponding *P* value between specific module and trait is recorded in each box. The color layout from blue to red indicates the correlative relationship from negative correlation to positive correlation. WGCNA, weighted correlation network analysis

To evaluate the clinical profiles of DEGs, WGCNA analysis was conducted to determine gene modules and correlation with clinical traits. Based on the optimal soft threshold, seven gene modules were identified and cluster dendrogram was obtained (Figure S2). The relationships between modules and clinical features including BCR status, BCR free survival, Gleason score, PSA level, clinical stage, and pathological T stage are shown in Figure 4. Of all the modules, three modules correlated strongly with BCR status (*P* < .05) and BCR free survival (*P* < .01), respectively. One module had a negative correlation with Gleason score (*P* < .05), while there was no significant correlation between gene modules and the other clinical factors. Based on the results, clinical prognosis was considered as the main association with DEGs. Prognosis analysis deserved to be conducted subsequently.

### 3.4 Prognostic analysis of DEGs

**FIGURE 5:**
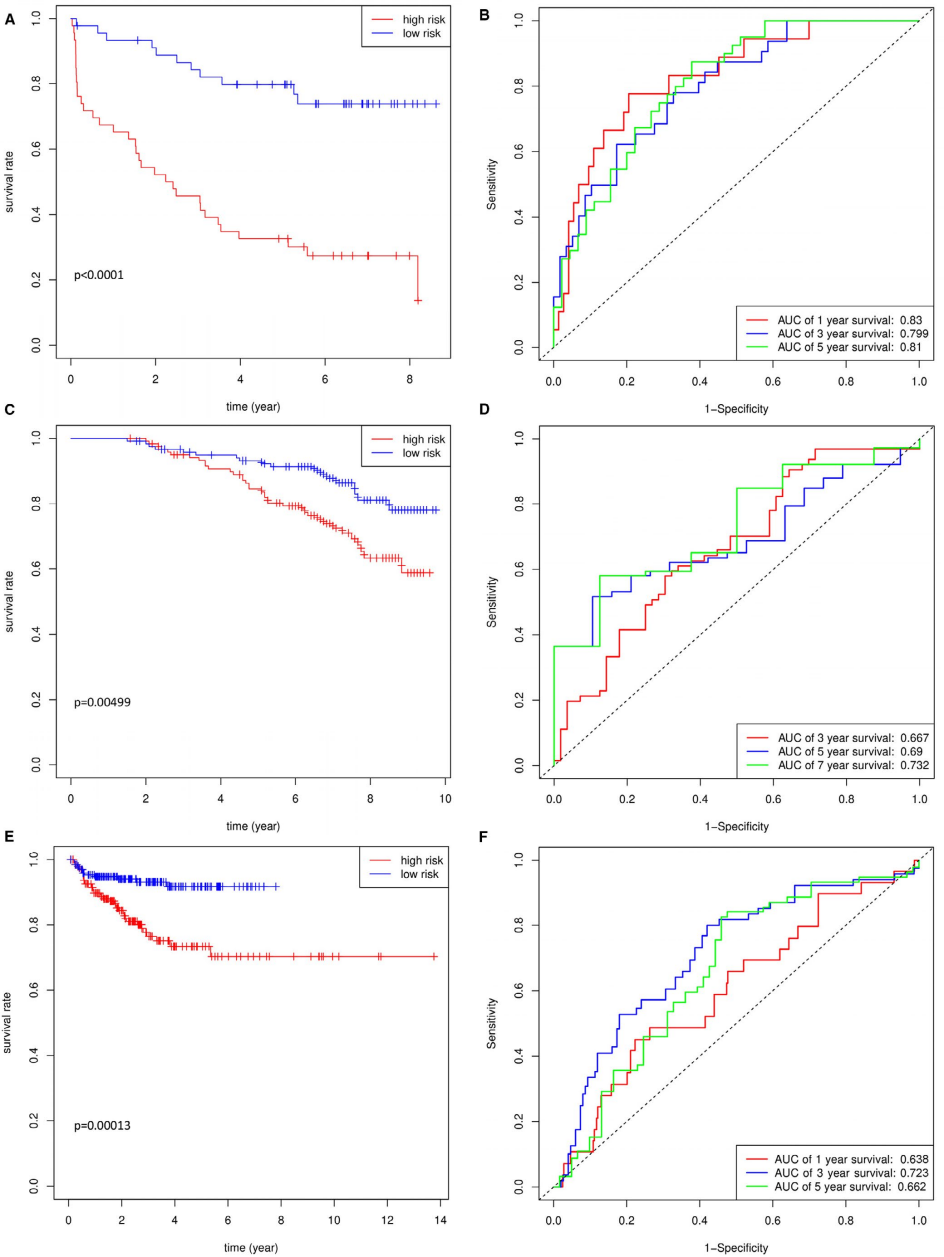
Prognosis analysis in training and validation datasets. Kaplan‐Meier BCR free survival curves for patients in GSE70769 (A), GSE106918 (C), and TCGA datasets (E) based on high‐ and low‐risk groups divided by risk score. ROC curve analyses of prognostic prediction for patients in GSE70769 (B), GSE106918 (D), and TCGA datasets (F). BCR, biochemical recurrence; ROC curve, receiver operational characteristic curve; TCGA, The Cancer Genome Atlas

To determine the prognostic value of DEGs, a univariate Cox regression was conducted to investigate the relationship between DEGs and BCR free survival in GSE 70 769 (Table S4). Then, 73 candidate DEGs were involved in LASSO Cox regression analysis and four effective DEGs were selected (Figure S3). Finally, ANO4, EZH2, PARM1, and SRD5A2 were regarded as prognostic genes and four‐gene prognostic model was constructed in stepwise multivariate Cox regression analysis. Risk scores for patients were calculated based on the integrated combination of gene expression level and corresponding regression coefficient. That is, risk score = (−1.25558 × expression of ANO4) + (1.337196 × expression of EZH2) + (−0.36793 × expression of PARM1) + (−0.73096 × expression of SRD5A2). Based on medium value of risk scores, patients in GSE70769 were divided into low‐ and high‐risk groups. Prognostic curves between BCR free survival and risk level were drawn (Figure 5A). It showed that low‐risk group had a better BCR free survival than that in high‐risk group (*P* < .0001). In the meantime, the AUCs of gene model corresponding to 1‐, 3‐, and 5‐year BCR free survival calculated based on ROC curves were 0.83, 0.799, and 0.81 (Figure 5B). It suggested good specificity and sensitivity of the four‐gene prognostic model.

### 3.5 External validation of four‐gene model in another GEO and TCGA datasets

To assess the external predictive effect of four‐gene model, clinical data in GSE116918 including 248 patients and TCGA dataset including 499 patients was analyzed. After calculating risk score for patients in GSE116918 and TCGA, patients were divided into low‐ and high‐risk groups using the four‐gene prognostic model. Consistent with prognostic information in GSE70769, BCR free survival was significantly better in the low‐risk group than that in high‐risk group in GSE116918 (*P* = .00499) and TCGA (*P* = .00013), respectively. These findings indicated the predictive effect of four‐gene model was well validated (Figure 5C‐F). In addition, we compared the predictive effect of four‐gene model with T stage in GSE70769. The patients were classified into different groups according the specific T stage. Patients with advanced T stage (T3 or higher) showed worse prognosis (*P* < .0001) compared with lower T stage. The AUCs of 0.816, 0.747, and 0.722 at 1, 3, and 5 years were similar with four‐gene model (Figure S4). Also, the C‐index for four‐gene model was 0.701(0.638, 0.764), which was comparable with T stage (0.708(0.634, 0.782)).

### 3.6 Validation of the expression of prognostic genes

**FIGURE 6:**
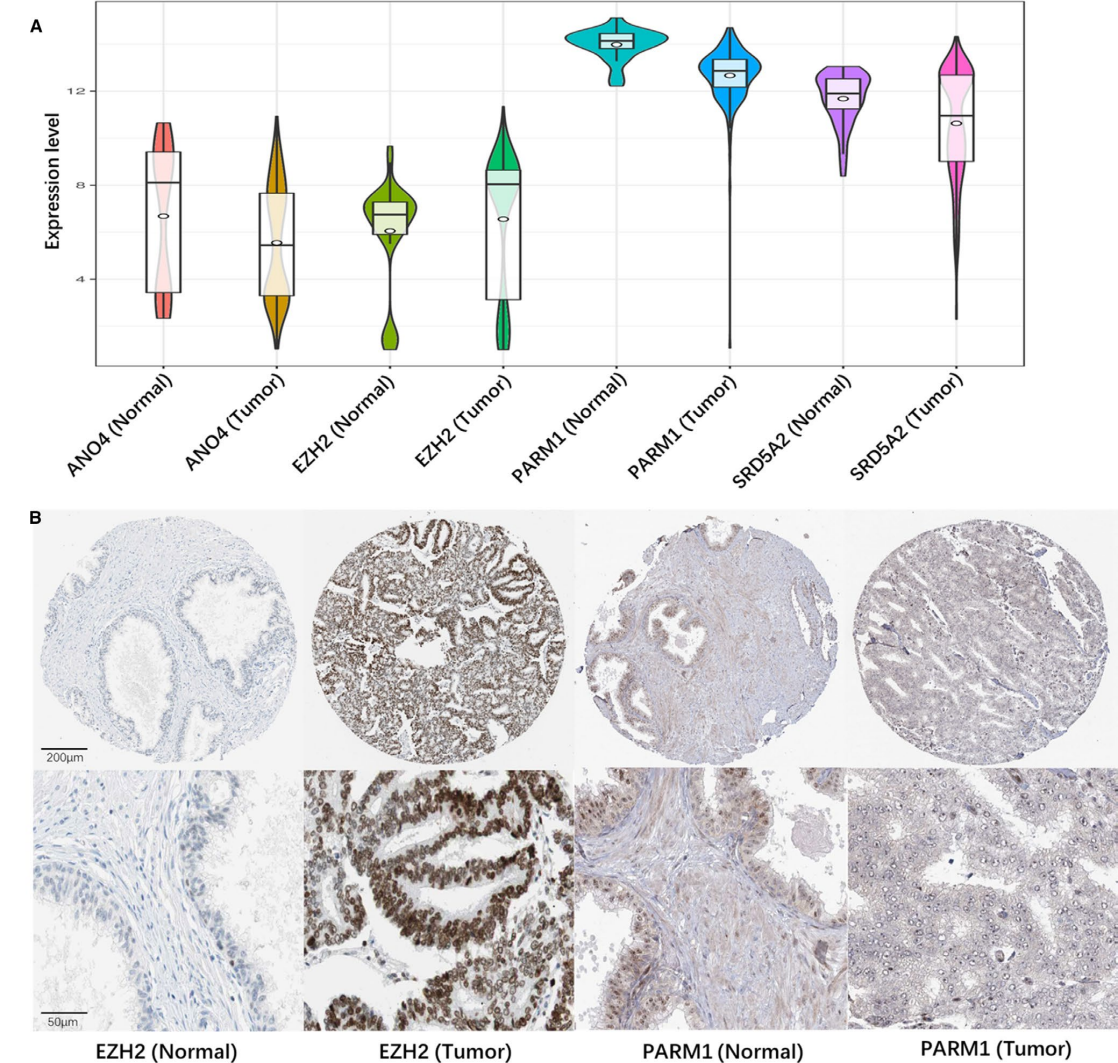
The expression profiles of prognostic genes in TCGA and HPA datasets. A, The expression levels after log2 transformation of the four genes in the TCGA prostate cancer RNA‐seq dataset. B, The immunohistochemical results of EZH2 and PARM1 in HPA. TCGA, The Cancer Genome Atlas; HPA, Human Protein Atlas; log2 transformation, base‐2 logarithm transformation; RNA‐seq, RNA sequencing

Besides external validation of prognostic prediction in GEO dataset, the expression patterns of prognostic genes were also assessed in TCGA and HPA datasets. As shown in TCGA (Figure 6A), the expression levels of ANO4 and PARM1 were significantly lower and EZH2 was significantly higher in PCa issue compared with normal issue (*P* < .05). What is more, in the HPA database, EZH2 was strongly positive in PCa issue and negative in normal tissue, while PARM1 was moderately positive in normal issue and negative in PCa tissue (Figure 6B). ANO4 and SRD5A2 were not found in the HPA database. All of the results were consistent with data in GEO datasets.

### 3.7 Assessment of independent predictive value of gene model

Four‐gene model and other clinical features including risk score, Gleason score, preoperative level of PSA, clinical stage, extra capsular extension, and positive surgical margins from GSE 70769 were involved in univariate Cox regression analysis first. It showed that risk score, Gleason score, extra capsular extension, and positive surgical margins had a relationship with prognosis (*P* < .05) (Figure S5). Then, these significant factors were incorporated into the multivariate Cox regression analysis. The result showed that risk score, Gleason score, extra capsular extension, and positive surgical margins had independent prognostic value associated with BCR free survival (Figure S5).

### 3.8 Construction and validation of a systematic prognostic model

**FIGURE 7:**
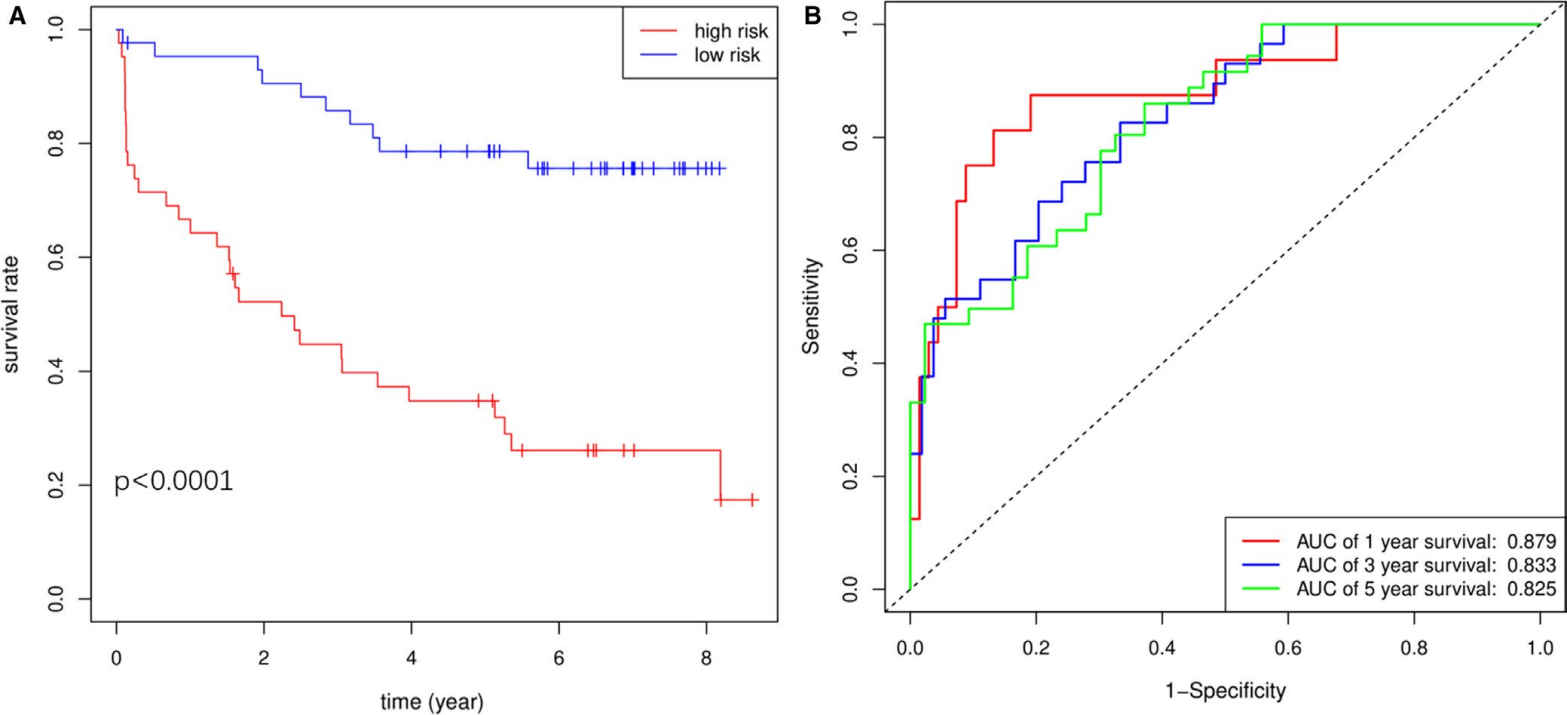
Prognosis analysis based on the combined model including four genes and clinical features. A, Kaplan‐Meier BCR free survival curves for patients in high‐ and low‐risk groups based on the combined model. B, ROC curve analyses of the combined model. BCR, biochemical recurrence; ROC curve, receiver operational characteristic curve

**FIGURE 8:**
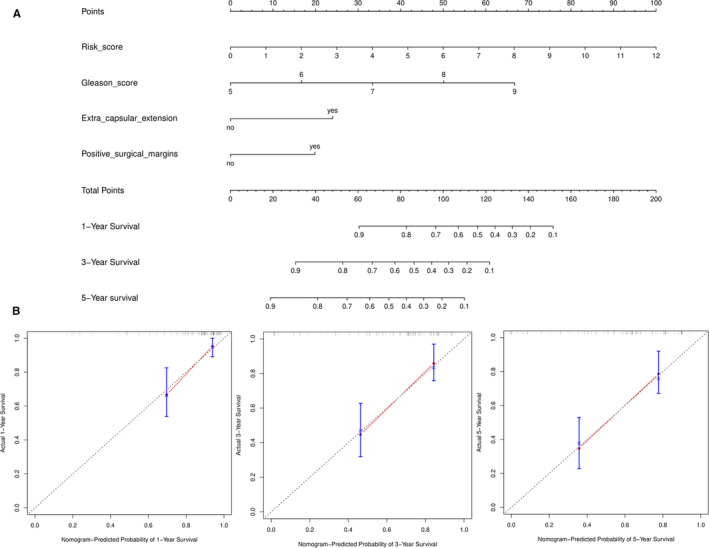
Nomogram evaluating prognosis based on the combined model. A, The 1‐, 3‐, and 5‐y BCR free survival could be evaluated by adding up the points of four‐gene risk score, Gleason score and events of extra capsular extension and positive surgical margins. B, The calibration curves for predicting 1‐, 3‐, and 5‐y BCR free survival for patients with prostate cancer

To construct a most effective prognostic model in PCa, risk score based on four genes and clinical features in multivariate Cox regression model were combined. The KM curves showed consistent results with four‐gene model (Figure 7A), but the AUCs of 0.879, 0.833, and 0.825 at 1, 3, and 5 years were higher (Figure 7B). The C‐index for the combined model was 0.732(0.668, 0.797). These results indicated better predictive value of combined model compared with T stage. Furthermore, a nomogram including risk score, Gleason score, extra capsular extension, and positive surgical margins was developed and the probability of recurrence free survival at 1, 3, and 5 years could be calculated based on these parameters (Figure 8A). Finally, calibration plots were drawn to reflect the accuracy of nomogram. Doublication degree between the solid line and dotted line showed the nomogram had a good predictive value (Figure 8B). In addition, the combined model based on four genes and clinical features was also successfully validated in another PCa cohort GSE70768 with 112 patients (Figure S6). The AUCs at 1, 3, and 5 years were 0.878, 0.768, and 0.778, respectively. The C‐index for the combined model was 0.688 (0.594, 0.782).

## 4 DISCUSSION

Prostate cancer is one of the most deadly urinary tumors in men globally.^1^ Study reported that there was 29 430 PCa‐related death cases in the United States in 2018.^29^ Therefore, it is of significant importance to expound biomarkers of PCa to achieve better diagnostic and prognostic evaluation. Overall survival and recurrence free survival are common indictors to assess the prognosis in cancers. In PCa, the emergence of BCR after radical prostatectomy and radiotherapy is confirmed as a decisive risk factor for PCa‐specific and overall mortality.^6^ Though some studies have researched gene expression profiles in PCa, systematic evaluation of molecular patterns as well as clinical information in PCa is limited.

To expound the potential biomarkers in PCa to a large extent, in the present study, we conducted an integrated analysis across multiple microarrays in GEO datasets. Significantly altered genes were identified based on robust rank aggregation.^30^ Then, a total of 270 DEGs involving 148 downregulated and 122 upregulated genes were obtained. GO enrichment analysis showed DEGs were significantly associated with 23 GO terms. Cell adhesion ^31^ and extracellular matrix ^32^ are well‐known components during the progression and metastasis of PCa. Peptidase activities also have a tight relationship with PCa. The kallikrein (KLK)‐related peptidase gene family plays an important role in the biological metabolism.^33^ KLK3 (PSA) is the most commonly used clinical marker for PCa at present.^34^ Besides, KLK2 and KLK4 are predominantly prostate specific and regulated by androgens.^35^ In KEGG analysis, DEGs mainly participated in Wnt signaling pathway, glutathione, and cytochrome P450 metabolism. Studies related to PCa have illustrated the underlying importance of molecular alterations in Wnt signaling pathway.^36^ In PCa microenvironment, Wnt‐related proteins derived from the tumor promote resistance to therapy.^37^ Besides, Wnt‐β catenin facilitates self‐renewing in PCa progenitors.^37^ Glutathione plays a pivotal role in the development of cancer.^38^ Glutathione metabolism is involved in the proliferation of PCa cells.^39^ As for cytochrome P450 metabolism, Chang et al^40^ reported that CYP1B1 inhibition suppressed tumorigenicity of PCa via caspase‐1 activation. And CYP17A1 was reported to be involved in the biosynthesis of androgen in human.^41^

To reveal the clinical profiles of DEGs, a co‐expression network by WCGNA was constructed between DEGs and clinical traits. Although only seven gene modules were obtained, three of them had a strong correlation with BCR status and BCR free time. These revealed the prognostic profile in DEGs. After univariate, LASSO and multivariate Cox regression analyses, ANO4, EZH2, PARM1, and SRD5A2 were screened out as prognostic genes ultimately. Then, a four‐gene prognostic model was constructed and patients were grouped based on risk score. The AUCs of the ROC curve for predicting the 1, 3, and 5‐year BCR free survival were 0.83, 0.799, and 0.81, respectively, indicating that the model had a good performance for prognostic prediction. Expression profiles of four genes in TCGA showed similar trend with GEO datasets. Furthermore, the predictive effect of the model was well validated in another PCa cohort and TCGA datasets.

Anoctamins, known as transmembrane 16 proteins, are a family of calcium‐activated chloride channels and involved in many biological processes, including membrane excitability, ion homeostasis, and cell proliferation.^42^ In cancer research, Britschgi et al^43^ found ANO1 promoted breast cancer progression by CAMK and EGFR pathways. Liu et al^44^ reported that upregulation of ANO1 is involved in the pathological process of metastatic PCa and inhibition of ANO1 is a promising method in PCa therapy. ANO4 colocalizes with the endoplasmic reticulum Ca^2+^‐ATPase and reduces Ca^2+^ store release, probably acting as a leakage channel.^45^ An analysis of single‐nucleotide polymorphisms in anoctamin genes showed that ANO4 gene expression had a protective effect on the prognosis of PCa,^46^ which is consistent with our results. However, the function of ANO4 in the pathological process of PCa is unknown, and molecular experiments need conducting to explore it further.

Enhancer of zeste homolog 2 (EZH2) is the enzymatic subunit of Polycomb repressive complex 2 related to transcriptional silencing.^47^ EZH2 alterations have been associated with cancer progression.^48^ EZH2 overexpression correlated with poor prognosis in tumors including prostate, breast, and bladder cancer.^49^ In PCa, EZH2 is reported to methylate the androgen receptor (AR) and modulate AR recruitment.^50^ After inhibiting EZH2, proliferation of PCa cells decreased and antitumor activity of AR antagonists in castration‐resistant PCa increased.^49^, ^51^ Also, EZH2 inhibition was powerful to prevent the progression of neuroendocrine PCa.^52^ Nowadays, EZH2 inhibitors are being evaluated in PCa patients such as CPI‐1205.^48^ It will be significant to improve the clinical outcome in PCa patients.

Prostate androgen‐regulated mucin‐like protein 1 (PARM1) is a member of the mucin family and is expressed at the surface of epithelial cells to promote cell survival.^53^ PARM1 was reported to promote cardiomyogenic differentiation through the Smad signaling pathway.^54^ It is also involved in the cancer development. PARM1 could suppress the proliferation of colorectal cancer cells.^55^ In PCa, Shola et al ^56^ found that PARM1 served as tumor suppressor to induce apoptosis of cancer cells through the Smad signaling pathway. In this study, downregulation of PARM1 showed a poor prognostic outcome in PCa patients and its expression was significantly lower in PCa issue compared with benign issue in TCGA and HPA datasets. However, it is also reported that PRAM1 enhanced cell growth in leukemia.^53^ The specific function of PARM1 in PCa need to be expounded further.

Steroid 5 alpha‐reductase 2 (SRD5A2) encodes a microsomal protein expressed at high levels in androgen‐sensitive tissues like prostate. In our study, SRD5A2 was regarded as a protective gene in the prognosis of PCa. SRD5A2 is involved in converting testosterone to dihydrotestosterone in prostate cells.^57^ Aggarwal et al^58^ reported that SRD5A2 could reduce cell migration and invasion by indirectly regulating ERK/MAPK pathway in PCa cells. Also, the expression of SRD5A2 was often found to be downregulated as androgen dependency is lost in advanced stages of metastasis.^59^ Furthermore, SRD5A2 polymorphism could be a promising biomarker for metastatic PCa patients treated with primary androgen‐deprivation therapy.^60^ Therefore, SRD5A2 may serve as a molecular target in the advanced PCa.

In a word, the four prognostic genes obtained in our study were well validated in theory and multiple datasets. It would be significant for the guidance of prognosis in PCa. Considering that traditional clinical parameters are also associated with prognosis, we combined four‐gene model and clinical factors to analyze the prognosis of PCa to a largest extent. Finally, we obtained a combined prognostic model including four genes and Gleason score, extra capsular extension, and positive surgical margins. Expectedly, the AUCs of the ROC curve for prognosis were higher than four‐gene model, which achieved a better predictive value. Based on the nomogram, prognostic probability for each patient could be predicted according to the corresponding parameters. A personalized evaluation was achieved in a sense.

There are some limitations in this study. First, the clinical data for prognostic analysis.

is not large enough, so prospective studies should be conducted further. Second, the specific mechanisms of four genes in PCa are need to be explored in molecular experiments.

In conclusion, we performed an integrated analysis in multiple microarrays and constructed a four‐gene prognostic model with ANO4, EZH2, PARM1, and SRD5A2 in PCa cohort. Then, the gene model was well validated in other datasets. Finally, combination of four‐gene model and clinical features achieved a better prediction to guide the prognosis of patients with PCa.

## CONFLICT OF INTEREST

No potential conflicts of interest are to be disclosed by authors.

## AUTHOR CONTRIBUTION

Conceptualization: PY and BL; Data curation and methodology: LL, XG, TS, JM, XY, and JL; Formal analysis: PY, LL, and QF; Funding acquisition: BL; Writing‐original draft: PY and BL; Writing‐review & editing: JM, XY, and JL; Supervision: JL and BL.

## ETHICAL APPROVAL

This study were approved by our Institutional Research Ethics Committee (Ethics Committee of Tongji Medical College, Huazhong University of Science and Technology).

## Supporting information

Fig S1‐S6

Table S1

Table S2

Table S3

Table S4

## Data Availability

The datasets involved in our study were extracted from TCGA ( https://portal.gdc.cancer.gov/), GEO (https://www.ncbi.nlm.nih.gov/geo), and HPA (https://www.proteinatlas.org/). All the data we used are available in our study.
