# Supplementary material for: A four‐gene signature associated with clinical features can better predict prognosis in prostate cancer
Source: Cancer Med. 2020 Sep 13;9(21):8202–15. doi: 10.1002/cam4.3453 (PMC7643642; doi:10.1002/cam4.3453)
Supplement: Supplementary file 1 — Fig S1‐S6 [file CAM4-9-8202-s001.docx]

**Supplementary Figures**

**Supplementary Figure 1**

A B

**
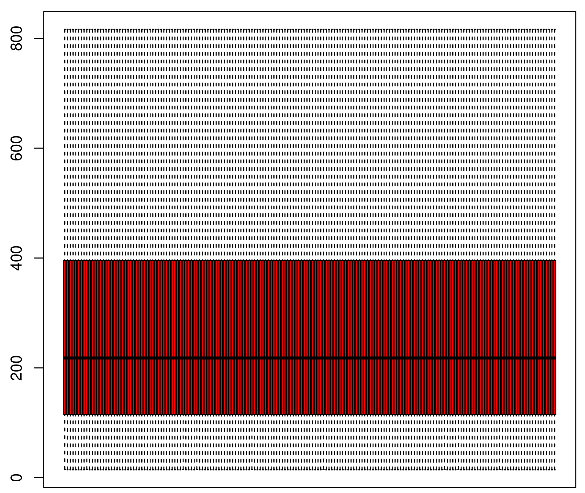

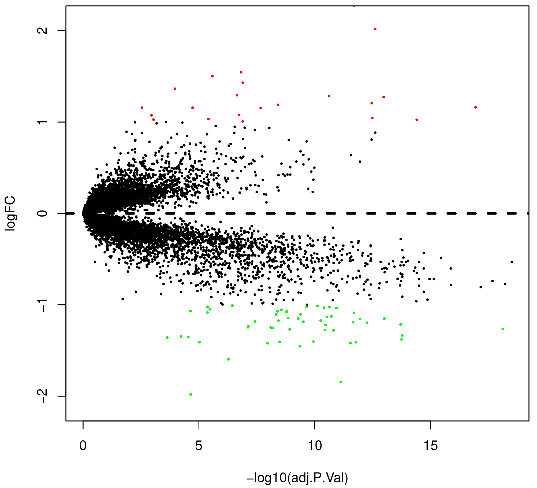
**

**Preprocessing and integrated analysis in each dataset.**

**(A) Normalization of data** **in GSE 21034. (B) Volcano plot of integrated analysis by the limma package in GSE 21034. The remaining datasets are not shown.**

**Supplementary Figure 2**


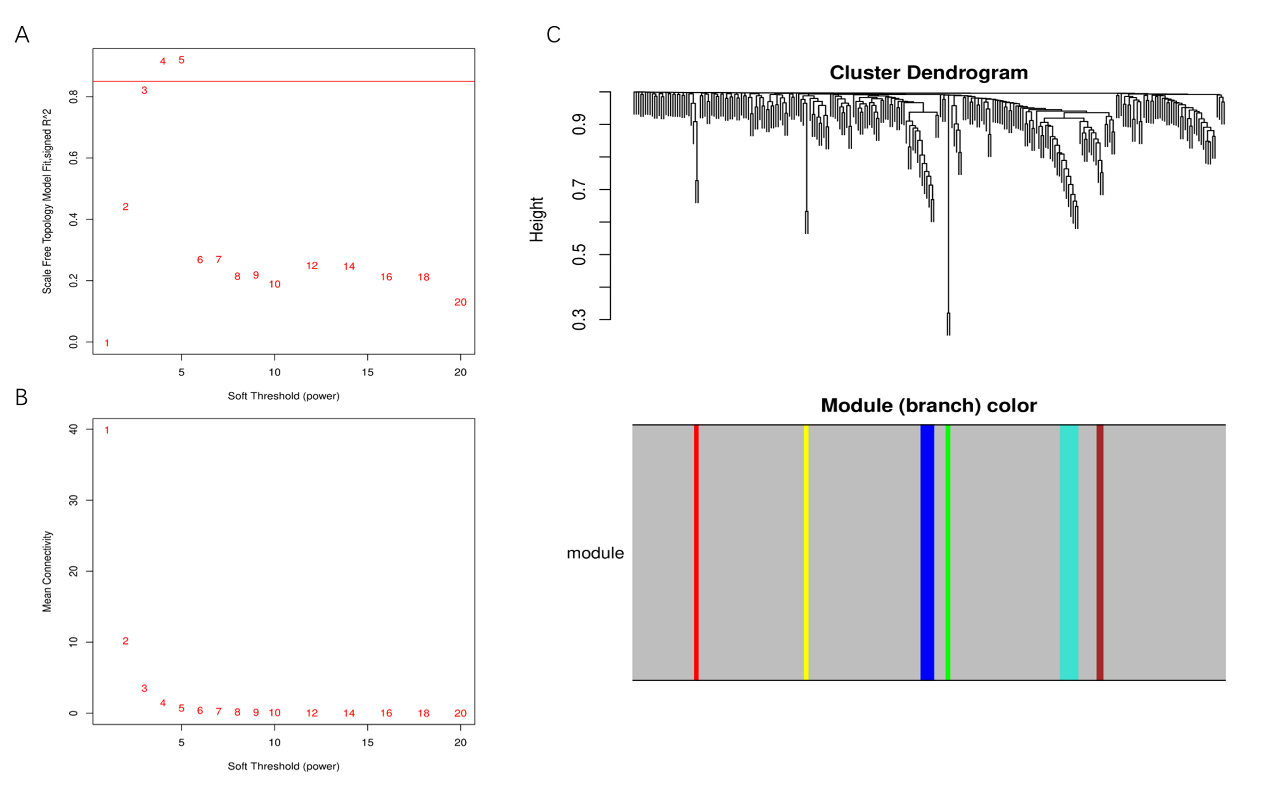


WCGNA analysis of DEGs.

(A) The relationship between scale free topology fit model index (scale free R^2^) and soft threshold. The red horizontal line represents R^2^ = 0.9. (B) The relationship between mean connectivity and soft threshold. (C) Clustering dendrogram of DEGs based on topological overlap measure.

WGCNA = weighted correlation network analysis; DEGs = differentially expressed genes.

**Supplementary Figure 3**


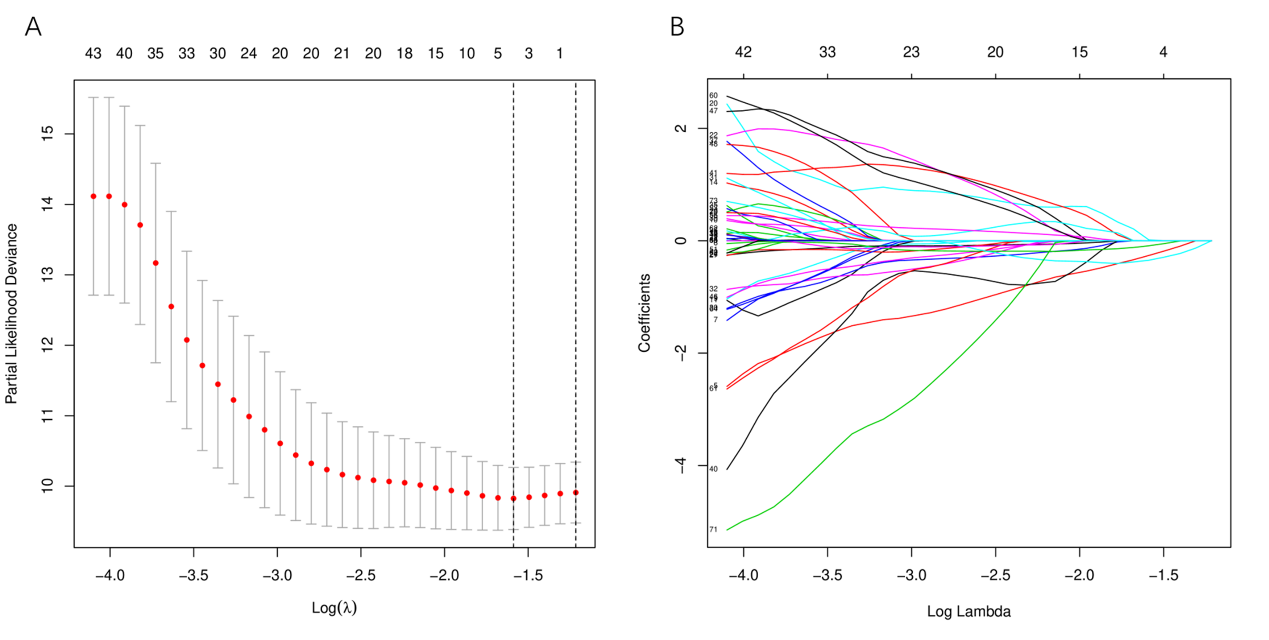


LASSO regression model.

(A) The relationship between partial likelihood deviance and tuning parameter. (B) The relationship between LASSO coefficients and tuning parameter. Each curve represents a coefficient.

**Supplementary Figure 4**


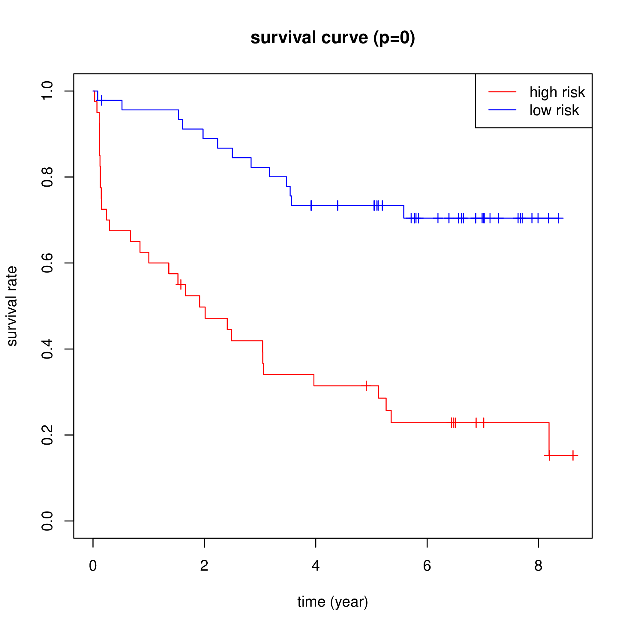

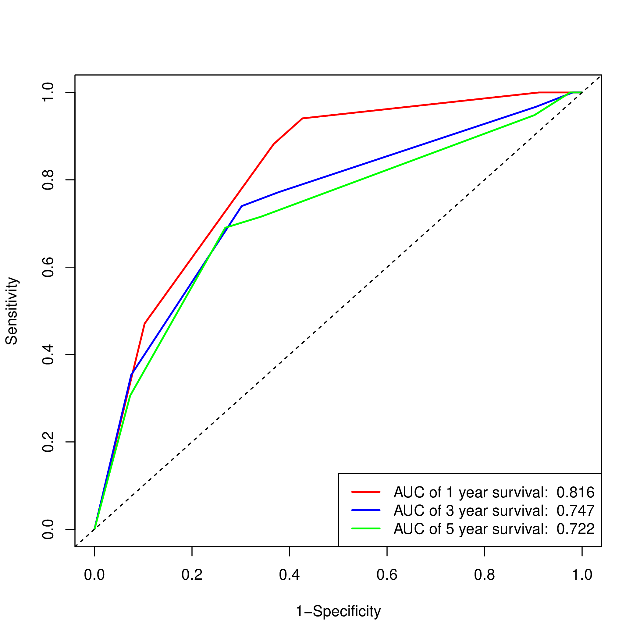


Prognosis analysis of T stage in PCa.

(A) Kaplan–Meier BCR free survival curve based on high- and low-risk groups divided by T stage. T3 or higher is regarded as high risk group and T2 or lower is regarded as low risk group. (B) ROC curve analysis of prognostic prediction for patients with PCa by T stage.

BCR = biochemical recurrence; ROC curve = receiver operational characteristic curve; PCa = prostate cancer.

**Supplementary Figure 5**


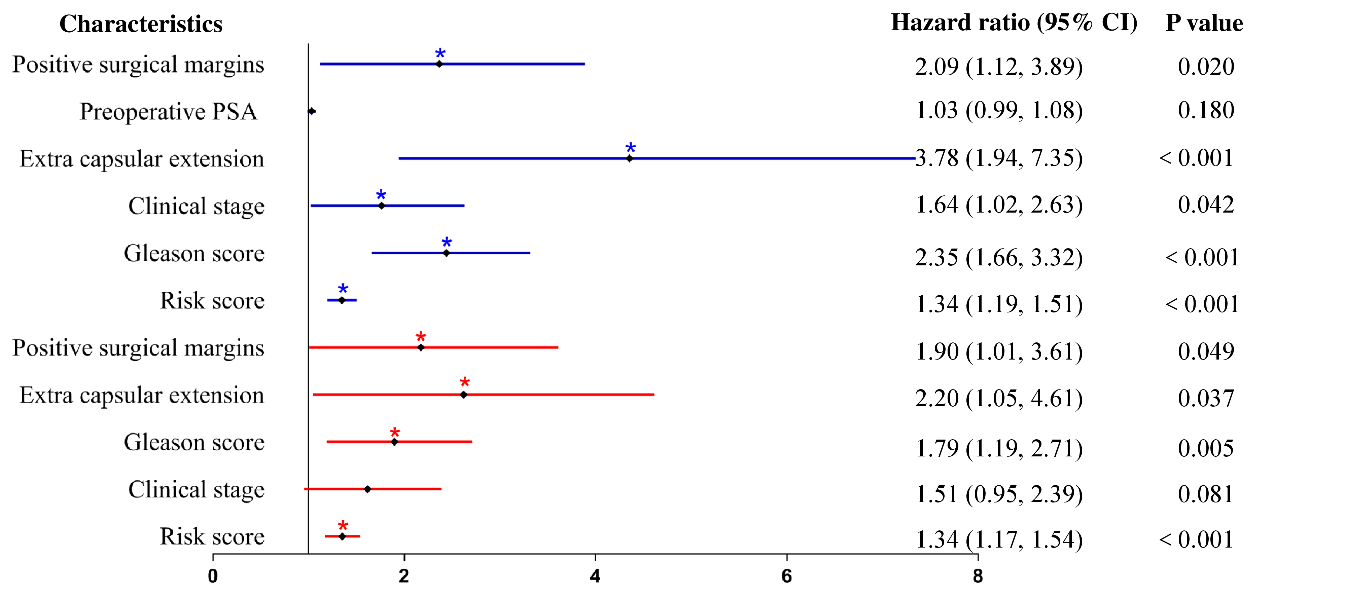


The results of univariate and multivariate Cox regression analyses between prognostic genes as well as clinical features and BCR free survival.

The factors involved in univariate and multivariate Cox regression models are represents by blue and red lines, respectively. Asterisk (*) represents statistical significance (p <0.05).

PSA = prostate-specific antigen; BCR = biochemical recurrence.

**Supplementary Figure 6**


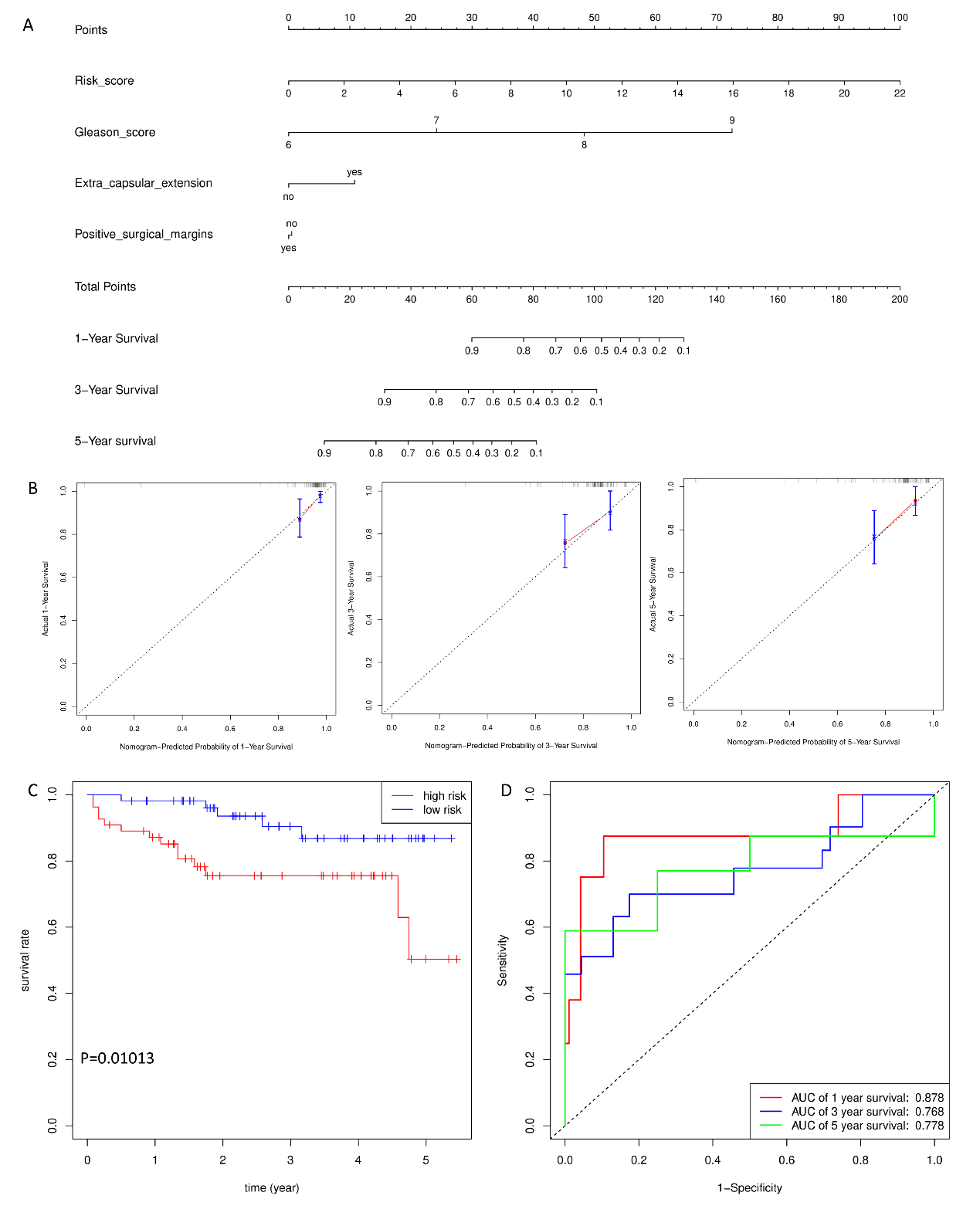


Validation of prognosis analysis based on the combined model including four genes and clinical features in GSE70768.

(A) Nomogram evaluating prognosis based on the combined model.

The 1-, 3- and 5-year BCR free survival could be evaluated by adding up the points of four-gene risk score, Gleason score and events of extra capsular extension and positive surgical margins. (B) The calibration curves for predicting 1-, 3- and 5-year BCR free survival for patients with prostate cancer in GSE70768. (C) Kaplan–Meier BCR free survival curves for patients in high- and low-risk groups based on the combined model. (D) ROC curve analyses of the combined model.

BCR = biochemical recurrence; ROC curve = receiver operational characteristic curve.
