# Supplementary material for: A four‐gene signature associated with clinical features can better predict prognosis in prostate cancer
Source: Cancer Med. 2020 Sep 13;9(21):8202–15. doi: 10.1002/cam4.3453 (PMC7643642; doi:10.1002/cam4.3453)
Supplement: Supplementary file 2 — Table S1 [file CAM4-9-8202-s002.docx]

**Supplementary Table 1**

Information of the five GEO datasets in this study.

| Dataset | Numbers of samples (Tumor/Control) | Platform | Last update date |
| --- | --- | --- | --- |
| GSE21034 | 179(150/29) | GPL10264 | Jul 10, 2014 |
| GSE29079 | 95(47/48) | GPL5175 | Feb 18, 2019 |
| GSE62872 | 424(264/160) | GPL19370 | Oct 08, 2019 |
| GSE46602 | 50(36/14) | GPL570 | Mar 25, 2019 |
| GSE55945 | 21(13/8) | GPL570 | Mar 25, 2019 |

GEO = Gene Expression Omnibus.
