# Supplementary material for: A four‐gene signature associated with clinical features can better predict prognosis in prostate cancer
Source: Cancer Med. 2020 Sep 13;9(21):8202–15. doi: 10.1002/cam4.3453 (PMC7643642; doi:10.1002/cam4.3453)
Supplement: Supplementary file 3 — Table S2 [file CAM4-9-8202-s003.docx]

**Supplementary Table 2**

GO enrichment analysis of DEGs

| ID | Description | p value | Count |
| --- | --- | --- | --- |
| GO:0004857 | enzyme inhibitor activity | p<0.0001 | 18 |
| GO:0050840 | extracellular matrix binding | p<0.0001 | 7 |
| GO:0004859 | phospholipase inhibitor activity | p<0.0001 | 4 |
| GO:1900750 | oligopeptide binding | p<0.0001 | 4 |
| GO:0055102 | lipase inhibitor activity | p<0.0001 | 4 |
| GO:0098641 | cadherin binding involved in cell-cell adhesion | 0.00013 | 4 |
| GO:0098631 | cell adhesion mediator activity | 0.00018 | 6 |
| GO:0072341 | modified amino acid binding | 0.00027 | 7 |
| GO:0030414 | peptidase inhibitor activity | 0.00028 | 10 |
| GO:0061134 | peptidase regulator activity | 0.0003 | 11 |
| GO:0043295 | glutathione binding | 0.00043 | 3 |
| GO:0016405 | CoA-ligase activity | 0.00046 | 4 |
| GO:0004364 | glutathione transferase activity | 0.00054 | 4 |
| GO:0005200 | structural constituent of cytoskeleton | 0.00062 | 7 |
| GO:0098632 | cell-cell adhesion mediator activity | 0.00069 | 5 |
| GO:0016878 | acid-thiol ligase activity | 0.00081 | 4 |
| GO:0015267 | channel activity | 0.00084 | 16 |
| GO:0022803 | passive transmembrane transporter activity | 0.00086 | 16 |
| GO:0004866 | endopeptidase inhibitor activity | 0.0009 | 9 |
| GO:0008238 | exopeptidase activity | 0.00115 | 7 |
| GO:0061135 | endopeptidase regulator activity | 0.00119 | 9 |
| GO:0003996 | acyl-CoA ligase activity | 0.00138 | 3 |
| GO:0008235 | metalloexopeptidase activity | 0.00185 | 5 |

GO = Gene Ontology; DEGs = differentially expressed genes.
