# Supplementary material for: A four‐gene signature associated with clinical features can better predict prognosis in prostate cancer
Source: Cancer Med. 2020 Sep 13;9(21):8202–15. doi: 10.1002/cam4.3453 (PMC7643642; doi:10.1002/cam4.3453)
Supplement: Supplementary file 4 — Table S3 [file CAM4-9-8202-s004.docx]

**Supplementary Table 3**

KEGG enrichment analysis of DEGs

| ID | Description | p value | Count |
| --- | --- | --- | --- |
| hsa00480 | Glutathione metabolism | p<0.0001 | 7 |
| hsa00982 | Drug metabolism - cytochrome P450 | 0.000107 | 7 |
| hsa05215 | Prostate cancer | 0.000684 | 7 |
| hsa00980 | Metabolism of xenobiotics by cytochrome P450 | 0.001115 | 6 |
| hsa05412 | Arrhythmogenic right ventricular cardiomyopathy | 0.001115 | 6 |
| hsa05204 | Chemical carcinogenesis | 0.001647 | 6 |
| hsa05410 | Hypertrophic cardiomyopathy | 0.002491 | 6 |
| hsa04310 | Wnt signaling pathway | 0.003088 | 8 |
| hsa05150 | Staphylococcus aureus infection | 0.003444 | 6 |
| hsa05414 | Dilated cardiomyopathy | 0.003444 | 6 |

KEGG = Kyoto Encyclopedia of Genes and Genomes; DEGs = differentially expressed genes.
