# Supplementary material for: A four‐gene signature associated with clinical features can better predict prognosis in prostate cancer
Source: Cancer Med. 2020 Sep 13;9(21):8202–15. doi: 10.1002/cam4.3453 (PMC7643642; doi:10.1002/cam4.3453)
Supplement: Supplementary file 5 — Table S4 [file CAM4-9-8202-s005.docx]

**Supplementary Table 4**

The results of univariate Cox regression between DEGs and BCR free survival.

| Gene | HR | z | p value |
| --- | --- | --- | --- |
| PAGE4 | 0.6233 | -4.3377 | p<0.0001 |
| SRD5A2 | 0.2118 | -4.2178 | p<0.0001 |
| PARM1 | 0.4181 | -4.0859 | p<0.0001 |
| PGM5 | 0.3725 | -4.0819 | p<0.0001 |
| EZH2 | 13.0763 | 3.8271 | 0.0001 |
| ANO4 | 0.0730 | -3.8021 | 0.0001 |
| TOP2A | 2.1257 | 3.6999 | 0.0002 |
| RCC1 | 55.6918 | 3.5490 | 0.0004 |
| MT1G | 0.7367 | -3.4547 | 0.0006 |
| TSPAN1 | 0.5155 | -3.3648 | 0.0008 |
| WIF1 | 5.1137 | 3.3447 | 0.0008 |
| MICAL2 | 4.8898 | 3.3361 | 0.0008 |
| HSPB8 | 0.3995 | -3.2563 | 0.0011 |
| GSTM5 | 0.1695 | -3.2485 | 0.0012 |
| GDF15 | 0.7101 | -3.2141 | 0.0013 |
| MEIS2 | 0.0435 | -3.1935 | 0.0014 |
| OR51E2 | 0.7793 | -3.1773 | 0.0015 |
| PHYHIPL | 9.0769 | 3.1370 | 0.0017 |
| CYFIP2 | 3.4152 | 3.0623 | 0.0022 |
| SMOC1 | 0.0369 | -2.9772 | 0.0029 |
| SCGB1A1 | 1.3627 | 2.7907 | 0.0053 |
| CNN1 | 0.6022 | -2.7860 | 0.0053 |
| AOX1 | 0.2525 | -2.7743 | 0.0055 |
| GOLM1 | 0.6113 | -2.7533 | 0.0059 |
| DPT | 0.4314 | -2.7339 | 0.0063 |
| NDRG2 | 0.3326 | -2.7143 | 0.0066 |
| BZW2 | 2.3456 | 2.6788 | 0.0074 |
| DES | 0.6010 | -2.6673 | 0.0076 |
| ITM2C | 0.5034 | -2.6671 | 0.0077 |
| SYNM | 0.3202 | -2.6573 | 0.0079 |
| BPHL | 0.4591 | -2.6533 | 0.0080 |
| GLYATL1 | 0.5651 | -2.6236 | 0.0087 |
| PUS7 | 2.7226 | 2.6075 | 0.0091 |
| CLIC6 | 0.3762 | -2.6033 | 0.0092 |
| CHRDL1 | 0.0169 | -2.5677 | 0.0102 |
| ACTC1 | 0.1933 | -2.5509 | 0.0107 |
| SERPINF1 | 0.5220 | -2.5410 | 0.0111 |
| LRCH2 | 0.5209 | -2.5294 | 0.0114 |
| WFDC2 | 0.0846 | -2.4707 | 0.0135 |
| TGFBR3 | 0.4457 | -2.4216 | 0.0155 |
| ANPEP | 0.7873 | -2.3988 | 0.0164 |
| TMC5 | 0.5724 | -2.3677 | 0.0179 |
| ANGPT1 | 0.3360 | -2.3155 | 0.0206 |
| ITGBL1 | 2.3937 | 2.2989 | 0.0215 |
| MAL2 | 1.9407 | 2.2874 | 0.0222 |
| SLC7A11 | 11.0747 | 2.2796 | 0.0226 |
| ABCC4 | 0.6999 | -2.2684 | 0.0233 |
| HPN | 0.4288 | -2.2526 | 0.0243 |
| OGN | 0.3337 | -2.2467 | 0.0247 |
| XPO6 | 2.1065 | 2.2326 | 0.0256 |
| JAZF1 | 0.3958 | -2.2272 | 0.0259 |
| NEK5 | 21.1150 | 2.2057 | 0.0274 |
| MSMB | 0.7913 | -2.1996 | 0.0278 |
| LMOD1 | 0.3371 | -2.1852 | 0.0289 |
| HLF | 0.2379 | -2.1847 | 0.0289 |
| MYL9 | 0.6180 | -2.1776 | 0.0294 |
| ALDH3B2 | 0.6153 | -2.1676 | 0.0302 |
| LGALS3 | 0.6257 | -2.1481 | 0.0317 |
| ITGA2 | 0.3686 | -2.1475 | 0.0318 |
| ENTPD5 | 0.6811 | -2.1193 | 0.0341 |
| BDH2 | 0.0878 | -2.1182 | 0.0342 |
| SPOCK3 | 0.2440 | -2.0981 | 0.0359 |
| DOPEY2 | 0.5026 | -2.0791 | 0.0376 |
| SPG20 | 0.1969 | -2.0381 | 0.0415 |
| GSTM2 | 0.5601 | -2.0144 | 0.0440 |
| FRMD6 | 0.3273 | -2.0093 | 0.0445 |
| SLC7A1 | 0.5842 | -1.9958 | 0.0460 |
| SCUBE2 | 1.2325 | 1.9958 | 0.0460 |
| SPDEF | 0.5096 | -1.9955 | 0.0460 |
| FLRT3 | 2.5506 | 1.9835 | 0.0473 |
| MPZL2 | 0.4169 | -1.9809 | 0.0476 |
| DNAJC10 | 0.5024 | -1.9784 | 0.0479 |
| F5 | 1.2189 | 1.9603 | 0.0500 |
| ACTG2 | 0.7440 | -1.9275 | 0.0539 |
| HOXD10 | 7.7794 | 1.9246 | 0.0543 |
| ATP8A2 | 12.0362 | 1.9161 | 0.0554 |
| FERMT2 | 0.4284 | -1.9070 | 0.0565 |
| GPM6B | 0.4384 | -1.8902 | 0.0587 |
| GUCY1A3 | 1.4955 | 1.8537 | 0.0638 |
| CYP27A1 | 0.7277 | -1.8518 | 0.0640 |
| BEX1 | 1.4507 | 1.8150 | 0.0695 |
| ASPN | 0.0477 | -1.7773 | 0.0755 |
| LSAMP | 1.5082 | 1.7637 | 0.0778 |
| TMEM45B | 1.3437 | 1.7616 | 0.0781 |
| STIL | 3.4185 | 1.7615 | 0.0782 |
| MYO6 | 1.3718 | 1.7604 | 0.0783 |
| CAMKK2 | 0.7168 | -1.7399 | 0.0819 |
| EFEMP1 | 2.0530 | 1.7275 | 0.0841 |
| CAV1 | 0.4677 | -1.7124 | 0.0868 |
| LEF1 | 1.4931 | 1.6936 | 0.0903 |
| MPPED2 | 0.5988 | -1.6912 | 0.0908 |
| TRHDE | 0.0743 | -1.6734 | 0.0942 |
| SLC27A2 | 0.6347 | -1.6518 | 0.0986 |
| EPCAM | 1.7847 | 1.6441 | 0.1002 |
| SLC18A2 | 13.3424 | 1.6271 | 0.1037 |
| KCNG3 | 1.5097 | 1.6083 | 0.1078 |
| NETO2 | 1.3903 | 1.6037 | 0.1088 |
| TCEAL2 | 0.6286 | -1.5977 | 0.1101 |
| SPP1 | 0.7184 | -1.5933 | 0.1111 |
| GSTM1 | 0.7093 | -1.5903 | 0.1118 |
| FABP5 | 0.7443 | -1.5656 | 0.1175 |
| PHF14 | 4.2590 | 1.5632 | 0.1180 |
| ASPA | 0.1890 | -1.5537 | 0.1203 |
| APOF | 0.7758 | -1.5502 | 0.1211 |
| NUP210 | 1.7812 | 1.5485 | 0.1215 |
| CREB3L1 | 0.1160 | -1.5207 | 0.1283 |
| ID4 | 3.2210 | 1.4942 | 0.1351 |
| REPS2 | 0.6306 | -1.4846 | 0.1376 |
| FRK | 0.4533 | -1.4618 | 0.1438 |
| SLC43A1 | 0.5654 | -1.4420 | 0.1493 |
| PPARGC1A | 0.3412 | -1.4403 | 0.1498 |
| ROR2 | 0.5280 | -1.4107 | 0.1583 |
| MYC | 1.3060 | 1.3960 | 0.1627 |
| PPP1R3C | 0.7015 | -1.3849 | 0.1661 |
| CAPG | 0.2723 | -1.3738 | 0.1695 |
| TP63 | 1.7466 | 1.3679 | 0.1713 |
| BICD1 | 2.3971 | 1.3185 | 0.1873 |
| SLC16A5 | 1.9353 | 1.3032 | 0.1925 |
| MGAT4A | 0.6561 | -1.3000 | 0.1936 |
| RND3 | 0.6717 | -1.2602 | 0.2076 |
| CPA6 | 1.8649 | 1.2533 | 0.2101 |
| GSTP1 | 0.6433 | -1.2450 | 0.2131 |
| PTGDS | 0.8251 | -1.2294 | 0.2189 |
| FBP1 | 0.7770 | -1.2054 | 0.2280 |
| HIST3H2A | 1.2927 | 1.1909 | 0.2337 |
| SFRP4 | 1.2771 | 1.1868 | 0.2353 |
| ACSM1 | 0.7693 | -1.1709 | 0.2416 |
| PLCB4 | 3.3678 | 1.1661 | 0.2436 |
| HSPB6 | 0.7515 | -1.1623 | 0.2451 |
| FNIP2 | 1.5470 | 1.1495 | 0.2503 |
| UAP1 | 0.7605 | -1.1420 | 0.2535 |
| CLIP4 | 0.5246 | -1.1097 | 0.2671 |
| HOXC6 | 1.8998 | 1.0976 | 0.2724 |
| PMM2 | 1.7205 | 1.0964 | 0.2729 |
| CACNA1D | 4.5887 | 1.0866 | 0.2772 |
| VWA5A | 0.5635 | -1.0725 | 0.2835 |
| ST6GALNAC2 | 0.5573 | -1.0723 | 0.2836 |
| CRYAB | 0.7229 | -1.0676 | 0.2857 |
| PAICS | 1.6249 | 1.0392 | 0.2987 |
| PRR16 | 1.1693 | 1.0368 | 0.2998 |
| LPAR1 | 0.4666 | -1.0279 | 0.3040 |
| CACNA2D1 | 0.2231 | -1.0226 | 0.3065 |
| ANXA2P2 | 0.4806 | -1.0080 | 0.3135 |
| PDGFC | 0.7040 | -1.0002 | 0.3172 |
| TRIB1 | 1.2142 | 0.9942 | 0.3201 |
| SNAI2 | 0.6986 | -0.9876 | 0.3234 |
| PTP4A3 | 0.7786 | -0.9649 | 0.3346 |
| FHL2 | 1.6732 | 0.9572 | 0.3385 |
| PCA3 | 0.9155 | -0.9543 | 0.3399 |
| COLEC12 | 0.6834 | -0.9206 | 0.3573 |
| TMLHE | 0.3108 | -0.9142 | 0.3606 |
| ACSS3 | 0.2435 | -0.8973 | 0.3696 |
| PALLD | 0.7508 | -0.8917 | 0.3726 |
| SPATA6 | 2.0903 | 0.8911 | 0.3729 |
| PLA2G7 | 1.0979 | 0.8655 | 0.3868 |
| STX19 | 0.7602 | -0.8528 | 0.3938 |
| TP53INP1 | 1.2113 | 0.8277 | 0.4079 |
| STEAP4 | 0.8383 | -0.8252 | 0.4093 |
| KRT19 | 0.8808 | -0.8163 | 0.4143 |
| PDE11A | 0.2165 | -0.7830 | 0.4336 |
| RAP1GAP | 1.1950 | 0.7762 | 0.4377 |
| HLA-DMB | 1.0931 | 0.7715 | 0.4404 |
| ANO5 | 3.5643 | 0.7699 | 0.4414 |
| TGFB3 | 0.8042 | -0.7651 | 0.4442 |
| CFL2 | 3.8727 | 0.7635 | 0.4452 |
| MYOF | 0.7882 | -0.7330 | 0.4635 |
| PDK4 | 1.1698 | 0.7253 | 0.4683 |
| KRT7 | 0.7362 | -0.7195 | 0.4718 |
| DNAJB4 | 0.4119 | -0.7124 | 0.4762 |
| PNMA1 | 1.3184 | 0.6952 | 0.4870 |
| GPRC5B | 2.2688 | 0.6908 | 0.4897 |
| GJA1 | 0.7837 | -0.6619 | 0.5080 |
| GDPD1 | 1.4258 | 0.6487 | 0.5165 |
| ID1 | 1.3017 | 0.6292 | 0.5292 |
| GJB1 | 0.8517 | -0.6252 | 0.5319 |
| CD177 | 1.3369 | 0.6229 | 0.5334 |
| SDK1 | 0.7749 | -0.5899 | 0.5552 |
| CHST9 | 0.8416 | -0.5861 | 0.5578 |
| PRDM8 | 1.1926 | 0.5832 | 0.5598 |
| TDRD1 | 0.8394 | -0.5606 | 0.5751 |
| TRIM29 | 1.7539 | 0.5482 | 0.5836 |
| CGREF1 | 0.6674 | -0.5466 | 0.5847 |
| ERG | 1.4230 | 0.5206 | 0.6026 |
| ANXA1 | 0.8972 | -0.5204 | 0.6028 |
| CYP3A5 | 0.6287 | -0.5107 | 0.6095 |
| FOLH1 | 1.0640 | 0.5050 | 0.6136 |
| TGM4 | 1.0659 | 0.4896 | 0.6244 |
| RALGAPA2 | 1.8113 | 0.4834 | 0.6288 |
| GCNT1 | 0.8950 | -0.4823 | 0.6296 |
| TFCP2L1 | 1.2727 | 0.4822 | 0.6297 |
| KRT15 | 0.9066 | -0.4787 | 0.6322 |
| GPR87 | 1.9338 | 0.4609 | 0.6449 |
| SERPINB5 | 0.7839 | -0.4589 | 0.6463 |
| SIM2 | 1.2096 | 0.4528 | 0.6507 |
| NRK | 0.6644 | -0.4399 | 0.6600 |
| S100A16 | 1.2657 | 0.4355 | 0.6632 |
| CRISP3 | 1.0654 | 0.4351 | 0.6635 |
| LUZP2 | 0.7581 | -0.4327 | 0.6653 |
| SLC14A1 | 1.2515 | 0.4285 | 0.6683 |
| CPAMD8 | 0.7272 | -0.4175 | 0.6763 |
| RAB3B | 0.9108 | -0.4149 | 0.6782 |
| GNAL | 2.5060 | 0.4136 | 0.6791 |
| CLDN8 | 1.0746 | 0.4099 | 0.6819 |
| KRT5 | 1.1400 | 0.4083 | 0.6830 |
| TMTC4 | 1.2510 | 0.3970 | 0.6914 |
| SERPINB1 | 0.8544 | -0.3947 | 0.6931 |
| SH3RF1 | 1.0931 | 0.3932 | 0.6942 |
| THBS4 | 1.0402 | 0.3659 | 0.7144 |
| CYP4B1 | 0.7177 | -0.3650 | 0.7151 |
| OLFM4 | 0.9523 | -0.3649 | 0.7152 |
| GABRB3 | 1.1498 | 0.3611 | 0.7180 |
| BEND4 | 1.1704 | 0.3600 | 0.7189 |
| DSC3 | 1.3041 | 0.3256 | 0.7447 |
| PI15 | 0.9629 | -0.3112 | 0.7556 |
| FGFR2 | 1.6044 | 0.3066 | 0.7592 |
| PLP2 | 0.8130 | -0.3004 | 0.7639 |
| GALNT7 | 0.7492 | -0.2952 | 0.7678 |
| ARMCX1 | 0.9193 | -0.2911 | 0.7710 |
| GPR160 | 1.0705 | 0.2733 | 0.7846 |
| PTPN13 | 1.0987 | 0.2724 | 0.7853 |
| SLITRK6 | 0.7505 | -0.2697 | 0.7874 |
| ACSF2 | 0.8056 | -0.2643 | 0.7916 |
| FADS2 | 1.0777 | 0.2639 | 0.7919 |
| NEFH | 1.0219 | 0.2538 | 0.7996 |
| MME | 1.0800 | 0.2360 | 0.8134 |
| RIPK2 | 1.0850 | 0.2277 | 0.8199 |
| FOLH1B | 1.0838 | 0.2077 | 0.8355 |
| EDNRB | 0.7729 | -0.2063 | 0.8366 |
| KRT13 | 1.0415 | 0.1940 | 0.8461 |
| MYOCD | 0.7273 | -0.1929 | 0.8470 |
| NR3C1 | 1.2848 | 0.1909 | 0.8486 |
| AMACR | 1.0233 | 0.1871 | 0.8516 |
| DNAH5 | 1.0973 | 0.1827 | 0.8550 |
| ANXA2 | 1.0760 | 0.1820 | 0.8556 |
| TSPAN13 | 1.0646 | 0.1819 | 0.8556 |
| FASN | 1.0330 | 0.1768 | 0.8597 |
| PPP1R14A | 1.0598 | 0.1651 | 0.8688 |
| SERPINB11 | 0.9731 | -0.1610 | 0.8721 |
| ZNF30 | 0.9671 | -0.1566 | 0.8756 |
| NAALADL2 | 1.1351 | 0.1563 | 0.8758 |
| BAMBI | 0.9770 | -0.1442 | 0.8853 |
| KCNH8 | 0.9203 | -0.1415 | 0.8875 |
| KRT23 | 0.8107 | -0.1317 | 0.8953 |
| CFH | 1.0570 | 0.1180 | 0.9061 |
| VSNL1 | 1.1340 | 0.1096 | 0.9127 |
| ANTXR2 | 1.0230 | 0.1044 | 0.9169 |
| LAMB3 | 1.1397 | 0.1041 | 0.9171 |
| IMPDH2 | 1.0257 | 0.0926 | 0.9262 |
| MAP2K6 | 0.8691 | -0.0821 | 0.9346 |
| LTF | 0.9899 | -0.0815 | 0.9350 |
| DAPK1 | 0.9698 | -0.0811 | 0.9354 |
| PLA1A | 0.9912 | -0.0780 | 0.9378 |
| GGCT | 0.9731 | -0.0744 | 0.9407 |
| GPX3 | 1.0107 | 0.0473 | 0.9623 |
| DNASE2B | 1.0090 | 0.0435 | 0.9653 |
| CENPN | 1.0056 | 0.0334 | 0.9733 |
| LMAN1L | 1.0076 | 0.0280 | 0.9776 |
| TRPC1 | 0.9858 | -0.0264 | 0.9790 |
| TRIM36 | 1.0095 | 0.0239 | 0.9810 |

DEGs = differentially expressed genes; BCR = biochemical recurrence; HR = hazard ratio.
